# Spatial Distribution and Determinants of Nonautonomy on Decision Regarding Contraceptive Utilization among Married Reproductive-Age Women in Ethiopia: Spatial and Bayesian Multilevel Analysis

**DOI:** 10.1155/2021/2160922

**Published:** 2021-11-05

**Authors:** Setognal Birara Aychiluhm, Kusse Urmale Mare, Mequannent Sharew Melaku, Abay Woday Tadesse

**Affiliations:** ^1^Department of Public Health, College of Medicine and Health Sciences, Samara University, Samara, Ethiopia; ^2^Department of Nursing, College of Medicine and Health Sciences, Samara University, Samara, Ethiopia; ^3^Department of Health Informatics, Institute of Public Health, College of Medicine and Health Sciences, University of Gondar, Gondar, Ethiopia; ^4^Dream Science and Technology College, Amhara Regional State, Dessie, Ethiopia

## Abstract

**Background:**

Studies conducted to date in Ethiopia did not explore the spatial distribution, individual-level, and community-level factors affecting women's nonautonomy on decision to use contraceptives. Hence, this study aimed to assess the spatial distribution of women's nonautonomy on decision regarding contraceptive utilization and its determinants in Ethiopia.

**Methods:**

Data were accessed from the Demographic Health Survey program official database website (https://dhsprogram.com). A weighted sample of 3,668 married reproductive-age women currently using contraceptives was included in this analysis. Bayesian multilevel logistic regression models were fitted to identify the determinants of women's nonautonomy on contraceptive utilization. Adjusted odds ratio with 95% credible interval was used to select variables that have a significant effect on nonautonomy on contraceptive utilization.

**Results:**

A high proportion of women with nonautonomy on decision regarding contraceptive utilization was found in northern parts of Southern Nations, Nationalities, and People's Region, Southern parts of Oromia, and Benishangul-Gumuz regions of the country. Overall, 2876 (78.40% (95% CI: 77.0%, 79.7%)) women were nonautonomous on decision regarding contraceptive utilization. In the final model, age from 35–49 (AOR (95% CI) = 0.63 (0.54, 0.72)), living in the richer households (AOR (95% CI) = 0.12 (0.03, 0.26)), being married at 18 years or above (AOR (95% CI) = 0.33 (0.19, 0.57)), and residing in an rural areas (AOR (95% CI) = 1.34 (1.01, 1.71)) and metropolitan regions (AOR (95% CI) = 0.71(0.54, 0.91)) were associated with women's nonautonomy on decision regarding contraceptive utilization.

**Conclusions:**

In Ethiopia, the spatial distribution of women's nonautonomy on decision about contraceptive utilization was nonrandom. More than three-fourths of married reproductive-age women in Ethiopia are nonautonomous on decision regarding contraceptive utilization. Region, residence, current age, age at marriage, and wealth index were statistically associated with women's nonautonomy on decision regarding contraceptive utilization.

## 1. Background

Although women's decision-making autonomy on sexual and reproductive health is crucial for better maternal and child health outcomes, restriction of open communication between partners due to gender-based power inequalities limits women's access to sexual and reproductive health services, particularly contraceptives [1]. The findings of different studies have shown the effects of women's autonomy on contraception utilization [2–4], and it is one of the influential sociocultural factors determining women's uptake of their preferred contraceptives [5–10]. In the settings where women are less autonomous on decision regarding contraceptive utilization, a low proportion of women use contraceptives [5, 11].

Globally, 45% of married women were nonautonomous on decision regarding SRH issues, with 64% in sub-Saharan Africa [12]. In Ethiopia, the existing evidence shows a considerable variation in the women's nonautonomy on decision regarding contraceptive utilization across different geographical areas that ranges from 20% to 78% [13–18]. Several studies conducted across the world have identified different community- and individual-level factors affecting women's nonautonomy on decision regarding contraceptive utilization [15–24].

Different strategies, policies, and programs have been strived in the past decades to improve safe motherhood at global, regional, and national levels [25]. In Ethiopia, promoting the use of SRH services and information as part of the reproductive health strategy [26], the incorporation of women's rights to information and rights to be protected from the risk of unwanted pregnancy through the use of contraceptive methods into the country's constitution [27], and implementation of five-year Health Sector Transformation Plan strategies [26, 28] were the efforts taken to improve reproductive health. Despite this, nearly 76% of women are nonautonomous on decision regarding contraceptive utilization in Ethiopia [21].

Studies conducted previously did not explore the spatial distribution of women's nonautonomy on decision regarding contraceptive utilization. Besides, individual- and community-level factors affecting women's nonautonomy on contraceptive utilization are not well investigated.

Identifying spatial distribution and determinants of women's nonautonomy helps to take targeted interventions and has become important to define geographical areas with women's nonautonomy on contraceptive utilization using Geographic Information Systems (GISs) and Spatial Scan Statistical (SaTScan) analyses. Besides, it could also be used as input for policymakers and programmer managers of the study area in the field of public health. Therefore, this study aimed to assess the spatial distribution of women's nonautonomy on decision regarding contraceptive utilization and its determinants in Ethiopia using Bayesian multilevel analysis.

## 2. Materials and Methods

### 2.1. Data Source, Study Period, Study Design, and Procedures

Data were retrieved from the Demographic and Health Survey (DHS) program official database website (https://dhsprogram.com), which were collected from January 18 to June 27, 2016. The Ethiopian Demographic and Health Survey (EDHS) is a nationally representative survey conducted every five years in the nine regional states (Afar, Amhara, Benishangul-Gumuz, Gambela, Harari, Oromia, Somali, Southern Nations, Nationalities, and People's Region, and Tigray) and two administrative cities (Addis Ababa and Dire-Dawa) of Ethiopia [29]. A total weighted sample of 3,668 married reproductive-age women currently using contraceptives was included in this study. The detailed sampling procedure exists in the full EDHS 2016 report [29].

### 2.2. Study Variables

#### 2.2.1. Dependent Variable

The outcome variable of this study was “women's autonomy on decision regarding contraceptive utilization.” The outcome variable was dichotomized into “nonautonomous = 1” and “autonomous = 0.”

#### 2.2.2. Independent Variables

Independent variables were classified into individual-level variables and community-level variables. Individual-level variables were the respondent's age, couple's age difference, marital status, type of marriage, women's education level, husband's education level, husband's occupation, respondent's occupation, wealth index, religion, exposure to mass media, age at marriage, and the number of living children. Community-level variables were region, residence, community media exposure, community women education level, and community poverty level. The community-level explanatory variables were constructed by aggregating individual-level characteristics at the community (cluster) level, and categorization of the aggregated variables was carried out as high or low based on the distribution of the proportion values calculated for each community.

### 2.3. Data Management and Statistical Analysis

We used ArcGIS version 10.6 and Spatial Scan Statistics (SaTScanTM version 9.6) software to perform the spatial data analysis. Global Moran's index (Moran's I) was used to measure spatial autocorrelation. Getis-Ord Gi^*∗*^ statistics were applied for hotspot analysis. Spatial scan statistics were applied to detect significant clusters. The scan statistics were developed using the Bernoulli model by applying by Kulldorff and SaTScan™ software version 9.6 to determine the presence of purely spatial nonautonomy on contraceptive utilization clusters.

Sample weights to the EDHS data were applied to estimate proportions and frequencies to adjust disproportionate sampling and nonresponse. A full clarification of the weighting procedure was explained in the 2016 EDHS report [29]. The analysis was performed using Stata version 16.0.

### 2.4. Convergence Assessment for Bayesian Multilevel Modeling

In this study, Markov Chain Monte Carlo (MCMC) simulation with Metropolis–Hastings sampling algorithm was carried out. To assess the convergence algorithm in our study, we used time-series (history) plots, density plots, and autocorrelation plots, and Gelman–Rubin statistics was used to assess whether the sample had reached stationary distribution or not.

### 2.5. Model Comparison and Selection

We have fitted four models that contain predictors of interest for this study: model I (null model), a model without independent variables to test random variability in the intercept and to estimate the intraclass correlation coefficient and proportion change in variance (PCV); model II, a model with only individual-level explanatory variables; model III, a model with only community-level explanatory variables; and model IV (full model), a model with both individual- and community-level predictors.

Deviance information criterion (DIC) value was used for model selection criteria, and the model with a low DIC value was considered as a more likely best-fitted model for this analysis. From the models fitted, model IV (full model), a model with both individual- and community-level predictors, has the smallest DIC value. Hence, model IV (full model) most likely fits the data.

Summary statistics were carried out from the posterior distribution, and adjusted odds ratio (AOR) with 95% Bayesian credible interval in the Bayesian multivariable multilevel analysis was used to select variables that have a statistically significant effect on women's nonautonomy on decision regarding contraceptive utilization.

### 2.6. Ethical Consideration

The data were accessed from the DHS website (https://www.measuredhs.com) after being registered and permission was obtained. The retrieved data were used for this registered research only. The data were treated as confidential, and no determination was made to identify any household or individual respondent.

## 3. Results

### 3.1. Sociodemographic Characteristics of the Study Participants

Out of the total respondents, 2,806 (76.5%) women resided in rural settings, 1,948 (53.1%) did not attend formal education, and 1,750 (47.7%) of the respondents' age ranged from 25–34 years. In this study, 1,988 (54.2%) of the study participants did not have exposure to mass media (Table 1).

### 3.2. Spatial Distribution of Nonautonomy on Contraceptive Utilization

**Figure 1:**
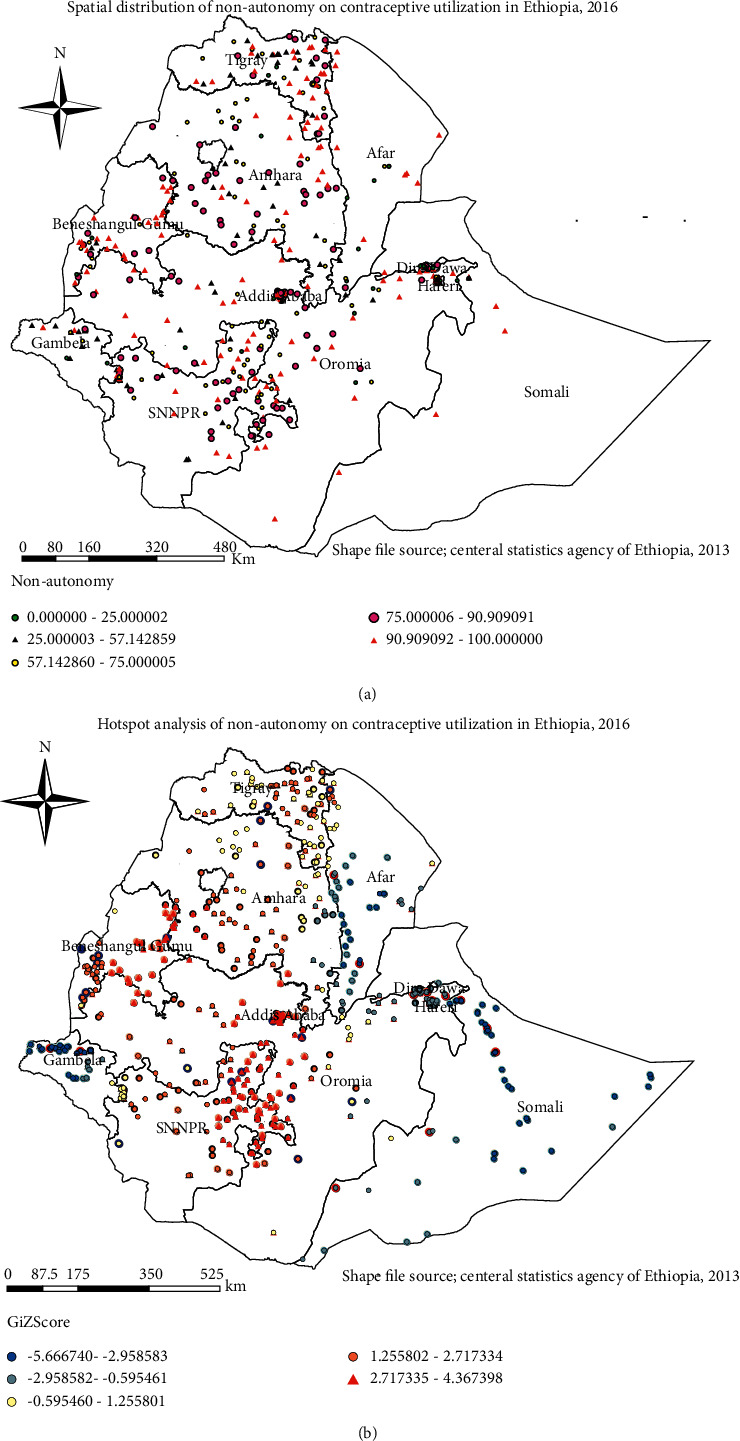
(a) The spatial distribution of women's nonautonomy on decision regarding contraceptive utilization, Ethiopia, 2016. (b) Hotspot analysis of women's nonautonomy on decision regarding contraceptive utilization, Ethiopia, 2016.

The spatial distribution of women's nonautonomy on contraceptive utilization in Ethiopia was nonrandom. The global Moran's I value was 0.082 (*P* value <0.001). Nonautonomous women on decision regarding contraceptive utilization were higher in Tigray, Amhara, eastern part of Afar, Eastern and Northern Somali, Benishangul-Gumuz, northern parts of SNNPR, Gambela, and Oromia regions. A low proportion of nonautonomous women was observed in Addis Ababa, Dire-Dawa, some parts of Amhara, Afar, Tigray, Gambela, and Oromia regions (Figure 1(a)).

### 3.3. Hotspot Analysis (Getis-Ord Gi^*∗*^Statistic)

High (hotspot) areas for nonautonomous women on contraceptive utilization were identified in northern parts of SNNPR, Southern parts of Oromia, and Benishangul-Gumuz regions which are represented in red color in Figure 1(b).

### 3.4. Spatial Scan Statistical Analysis

**Figure 2:**
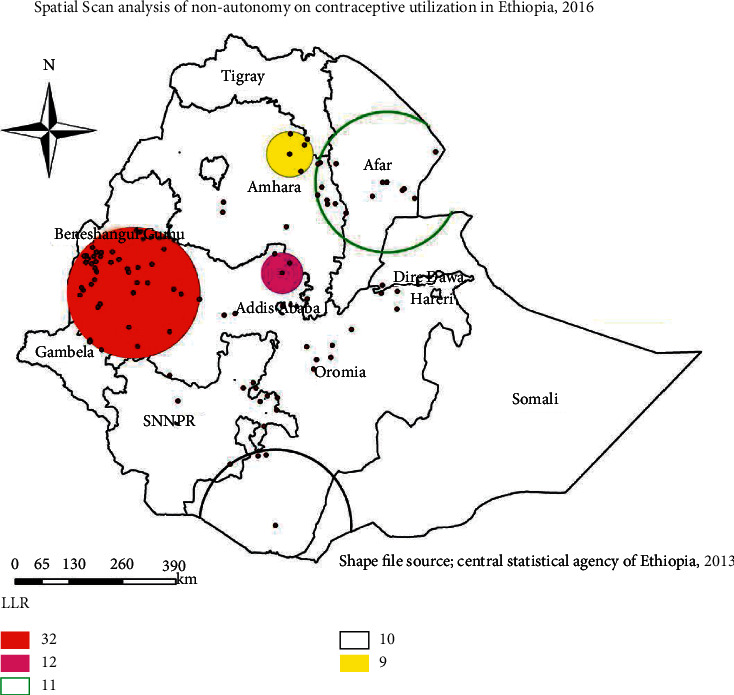
Spatial scan statistics of women's nonautonomy on decision regarding contraceptive utilization, Ethiopia, 2016.

In spatial scan statistics, 1 primary and 4 secondary clusters were identified; the primary cluster is located at 9.306441 N, 35.546886 E with a 159.63 km radius, a Relative Risk (RR) of 1.23, and an LLR of 32.11 (Figure 2 and Table 2). It showed that women inside the spatial window had 1.23 times higher likelihood of being nonautonomous on decision regarding contraceptive utilization than women outside the spatial window. The most likely clusters of nonautonomous women were detected in most parts of Benishangul Gumz and western parts of Oromia region.

### 3.5. Women's Nonautonomy on Decision Regarding Contraceptive Utilization

Overall, 2876 (78.40% (95% CI: 77.0%, 79.7%)) women were nonautonomous on contraceptive utilization.

### 3.6. Result of the Empty (Null) Bayesian Multilevel Logistic Regression Model

The Bayesian null model showed that variance of the random part was 0.46 with a 95% credible interval of 0.22–0.71, showing EA differences in women's nonautonomy on contraceptive utilization in Ethiopia. The variance estimate, which is greater than zero, indicates that there are EA, differences in nonautonomy on contraceptive utilization among married reproductive-age women in the country.

The unobserved heterogeneity has a logistic distribution with a variance at the individual level equivalent to ∏^2^/3 (that is, 3.29) [30–32]. Therefore, the ICC = 0.46/0.46 + 3.29 = 0.12, which implied that 12% of the total variability in nonautonomy on decision regarding contraceptive utilization among married reproductive-age women is due to differences across enumeration areas, and 88% of the variability is accounted by individual differences. Both the random factor variance and the ICC value were proposed to apply the Bayesian multilevel logistic regression model for additional analysis to handle the heterogeneity between EAs (Table 3).

### 3.7. Bayesian Multilevel Logistic Regression Analysis

The random-walk Metropolis–Hastings sampling procedure was applied with 12,500 total iterations. After 2,500 burn-in terms were discarded, 10,000 samples were generated from the full posterior distribution. Noninformative normal prior distribution with mean = 0 and variance = 10^6^ for the fixed-effect and gamma distribution with scale = 0.1 and shape = 0.1 for the variance of random effect was used. Convergence-assessment plots have confirmed the convergence produced from Markov chains, before taking any inference from the posterior distribution. Before undertaking any inference from the posterior distribution, the convergence generated from Markov chains was proved by convergence-assessment plots (Annex 1)

### 3.8. Bayesian Multivariable Multilevel Logistic Regression Model

In the Bayesian multivariable multilevel logistic regression model, region, residence, current age, age at first marriage, and wealth index were statistically associated with women's nonautonomy on decision about contraceptive utilization. After adjusting for covariates, the odds of being nonautonomous on contraceptive utilization among women residing in rural settings was 51% lower than that of those living in an urban area (AOR (95% CI) = 1.34 (1.01, 1.71)).

Women who had their first marriage at the age of 18 years and above were 67% less likely to be nonautonomous on decision to use contraceptives compared to those married before 18 years (AOR (95% CI) = 0.33 (0.19, 0.57)). Similarly, women in the age range of 35–49 years (AOR (95% CI) = 0.63 (0.54, 0.72)) had lesser odds of being nonautonomous on decision regarding contraceptive utilization compared to younger women.

Our analysis also revealed that the likelihood of being nonautonomous on decision regarding contraceptive utilization was 82% lower for women in rich households compared to those from poor households (AOR (95% CI) = 0.12 (0.03, 0.26)). Moreover, women living in metropolitan regions were less likely to be nonautonomous on decision regarding contraceptive utilization compared to those in large central regions (AOR (95% CI) = 0.71(0.54, 0.91)) (Table 4).

## 4. Discussion

Women's decision-making autonomy on contraceptive utilization is an essential component of SRH rights [33]. This study assessed the spatial distribution and determinants of nonautonomy on decision regarding contraceptive utilization among married women in Ethiopia. It was revealed that more than three-fourth women in Ethiopia were nonautonomous on decision regarding contraceptive utilization. This finding is higher compared to the results of the studies in different parts of Ethiopia [15–19, 34] and South Africa [35]. On the contrary, our finding is lower than in a study in Senegal [36]. The discrepancy might be due to the methodological differences of the studies and variations in the sociocultural and religious context of the study areas.

The spatial analysis showed that, in Ethiopia, the spatial distribution of women's nonautonomy on contraceptive utilization was nonrandom. The clustered pattern was confirmed with hotspot and spatial SaTScan analysis.

Region, residence, wealth index, current age, and age at first marriage were identified as significant factors affecting women's nonautonomy on decision about contraceptive utilization. Accordingly, women from rural areas had increased likelihood of being nonautonomous on contraceptive utilization compared to those residing in urban settings. This finding is consistent with the result of the previous studies in Ethiopia [20, 21], which found lower odds of nonautonomy among urban women. This might be because women in urban residences have better educational opportunities and have access to information, particularly on contraceptives and other SRH-related issues, than their rural counter group, which enables them to have greater involvement in contraceptives and other household decision-making processes.

Women aged 35–49 years were less likely to be nonautonomous on decision regarding contraceptive utilization than women aged 15–24 years. This finding is similar to the previous studies in Ethiopia [19, 21, 36]. This might be because younger women are less likely to visit family-planning clinics and lack awareness due to limited access to SRH information [37] and, therefore, have little control over their contraceptive decision. On the contrary, this finding is inconsistent with studies in Southern Ethiopia [16, 18]. Methodological differences might contribute to these variations.

Women who had their first marriage after the age of 18 years and above had decreased odds of being nonautonomous compared to those married before 18 years. This finding is consistent with a previous study [12]. This might be due to the inferior negotiating power of younger women associated with limited educational opportunities as a consequence of early marriage [38, 39].

This study also showed that women who lived in metropolitan regions had decreased likelihood of being nonautonomous on decision regarding contraceptive utilization compared to those in large central regions. Differences in urbanization and access to sexual and reproductive health services and its information which have a direct relation with healthcare decision might have contributed to this finding [22, 40].

Furthermore, consistent with the results of previous studies [12, 41], this study revealed that women from rich households had lesser odds of being nonautonomous on a decision regarding contraceptive utilization compared to those from poor households. This might be because women in the richer household are more likely to be employed, have an increased level of self-confidence, and have access to information, which could improve their involvement in healthcare decisions [42].

This study has strengths of nationally representative weighted data, and appropriate advanced statistical models were used to account for the clustering effect and to get a reliable standard error and parameter estimates. Moreover, the use of GIS and SaTScan statistical tests helps to detect similar and statistically significant hotspot areas of nonautonomy on decision regarding contraceptive utilization. However, this study has limitations of the cross-sectional nature of the study, which may not indicate true causality.

Public health interventions targeting significant hotspot areas are essential to enhance the autonomy of women on contraceptive utilization. Moreover, the government should promote women's autonomy on contraceptive utilization as an essential component of SRH with particular attention for adolescent women, women living in the poorest households, and those residing in rural settings of the country.

The findings of this study have valuable policy implications for health programme design and interventions. High-risk areas for nonautonomy on contraceptive utilization can be easily identified to make effective local interventions. In general, these findings are of importance for the minister of health, regional health bureaus, and nongovernmental organizations when designing an intervention to reduce nonautonomy on decision on contraceptive utilization in hotspot areas identified by the study.

## 5. Conclusions

More than three-fourths of married reproductive-age women in Ethiopia were nonautonomous on decision regarding contraceptive utilization. A high (hotspot) proportion of women with nonautonomy was found in northern parts of SNNPR, southern parts of Oromia, and Benishangul-Gumuz regions of the country. Region, residence, age, age at first marriage, and wealth index were statistically associated with women's nonautonomy on decision about contraceptive utilization.

## Figures and Tables

**Table 1:** Weighted sociodemographic characteristics of the study participants, Ethiopia, 2016.

Variables	Frequency (%)
Residence	
Urban	8629 (23.50)
Rural	2,806 (76.50)
Religion	
Orthodox	1,877 (51.17)
Protestant	968 (26.38)
Muslim	768 (20.94)
Others^+^	55 (1.51)
Age (years)	
15–24 years	850 (23.18)
25–34 years	1,750 (47.70)
3–49 years	1,068 (29.12)
Age at first marriage	
<18 years	1,397 (38.08)
≥18 years	2,271 (61.92)
Respondent's educational status	
No education	1,948 (53.10)
Primary	1,146 (31.25)
Secondary and above	574 (15.65)
Husband's educational status	
No education	1,427 (38.91)
Primary	1,445 (39.39)
Secondary and above	796 (21.70)
Respondent's occupation	
Not employed	1,844 (50.27)
Employed	1,824 (49.73)
Husband's occupation	
Not employed	1,844 (50.27)
Employed	1,824 (49.73)
Wealth index	
Poor	1,028 (28.02)
Middle	765 (20.85)
Rich	1,875 (51.14)
Media exposure	
No	1,988 (54.20)
Yes	1,680 (45.80)
Number of living children	
<=2	1,635 (44.56)
>2	2,033 (55.44)
Type of marriage	
Monogamy	3,497 (95.3)
Polygamy	171 (4.7)
Couple's age difference	
Negative	119 (3.2)
Equal	79 (2.2)
≤10 years	2,767 (75.4)
≥10 years	703 (19.2)

Others^+^ = catholic, traditional, and other EDHS categories. Others^++^ = other EDHS categories. Others^+++^ = other EDHS categories.

**Table 2:** The spatial scan statistical analysis of women's decision-making nonautonomy on contraceptive utilization, Ethiopia, 2016.

Cluster type	Number of significant enumeration areas	Coordinates/radius	Populations	Cases	RR	LLR	*P* value
1	53	(9.306441 N, 35.546886 E)/159.63 km	272	259	1.23	32.11	<0.001
2	3	(9.739794 N, 38.793594 E)/49.89 km	65	64	1.26	11.96	0.003
3	17	(11.731000 N, 41.095173 E)/170.63 km	100	95	1.22	11.01	0.010
4	4	(4.211065 N, 38.646702 E)/183.90 km	40	40	1.28	9.72	0.021
5	5	(12.349051 N, 38.961666 E)/55.64 km	37	37	1.28	8.98	0.044

**Table 3:** Estimates for the variance components model of women's decision-making nonautonomy on contraceptive utilization, Ethiopia, 2016.

Fixed effect Intercept Random effect	Estimate 3.38 Estimate	SDMCSE 0.201 SD	0.013	95% credible interval (3.01, 3.79) 95% credible interval
*δ*_*u*_^2^	0.46	0.12	0.016	(0.22, 0.71)
ICC	0.12	—		—

**Table 4:** Bayesian multilevel multivariable logistic regression of the individual- and community-related variables associated with women's nonautonomy on contraceptive utilization, Ethiopia, 2016.

Variables	Women's autonomy						
	No	Yes	SD	MCSE	Model II AOR (95%)	Model III AOR (95%)	Model IV AOR (95%)
Age (years)							
15–24 years	600 (78.7)	162 (21.3)	—	—	1	—	1
25–34 years	1040 (76.1)	326 (23.9)	0.077	0.009	0.74 (0.60,0.90)^*∗*^		0.85 (0.68, 1.03)
35–49 years	614 (72.1)	238 (27.9)			0.48 (0.36,0.61)^*∗*^		0.63 (0.54, 0.72)^*∗*^
Religion							
Orthodox	1189 (75.0)	397 (25.0)				—	1
Protestant	512 (80.3)	126 (19.7)	0.123	0.015	1.32 (1.10,1.57)^*∗*^		0.89 (0.58, 1.37)
Muslim	523 (72.6)	197 (27.4)	0.065	0.012	0.81 (0.69,0.95)^*∗*^		0.62 (0.42, 1.95)
Others^+^	30 (83.3)	6 (16.7)	0.154	0.023	1.53 (1.25,1.85)^*∗*^		0.56 (0.18, 1.22)
Age at 1^st^ marriage							
<18 years	1317 (76.1)	414 (23.9)	—				1
≥18 years	937 (75.0)	312 (25.0)	0.084	0.016	0.48 (0.22,0.65)^*∗*^		0.33 (0.19,0.57)^*∗*^
Respondent's educational status							
No education	954 (75.2)	315 (24.8)					—
Primary	792 (76.5)	244 (23.5)	0.074	0.006	1.03 (0.89,1.18)		
Secondary and above	508 (75.3)	167 (24.7)	0.09	0.015	1.09 (0.91,1.29)		
Husband's educational status							
No education	683 (74.1)	239 (25.9)					—
Primary	888 (77.8)	253 (22.1)	0.732	0.012	1.05 (0.91,1.20)		
Secondary and above	683 (74.5)	234 (25.5)	0.108	0.024	0.90 (0.72,1.13)		
Husband's occupation							
Unemployed	1121 (74.5)	384 (25.5)					—
Employed	1133 (76.8)	342 (23.2)	0.072	0.009	1.20 (1.06,1.36)		
Wealth index							
Poor	532 (74.5)	182 (25.5)					1
Middle	368 (78.8)	99 (21.2)	0.063	0.011	0.32 (0.09,1.56)		0.21 (0.01,1.10)
Rich	1354 (75.3)	445 (24.7)	0.078	0.014	0.23 (0.05,0.44)^*∗*^		0.12 (0.03,0.26)^*∗*^
Media exposure							
No	1016 (77.1)	301 (22.8)					—
Yes	1238 (74.4)	425 (25.6)	0.099	0.014	0.89 (0.72,1.11)		
Number of living children							
≤2	1154 (75.2)	380 (24.8)					1
>2	1100 (76.1)	346 (23.9)	0.130	0.014	1.14 (1.19,1.72)^*∗*^		1.32 (0.98, 1.62)
Residence							
Urban	803 (71.2)	325 (28.8)				1	1
Rural	1451 (78.3)	401 (21.6)	0.35	0.024		1.61 (1.06,2.45)	1.34 (1.01, 1.71)^*∗*^
Region							
Large central	1357 (77.5)	393 (22.5)			—	1	1
Small peripheral	408 (79.5)	105 (20.5)	0.195	0.029		1.13 (0.79,1.56)	1.16 (0.92,1.44)
Metropolitan	489 (68.2)	228 (31.8)	0.107	0.012		0.62 (0.42,0.86)	0.71 (0.54,0.91)^*∗*^
Community media exposure							
Low	1149 (77.8)	327 (22.2)	0.167	0.022	—	1	—
High	1105 (73.5)	399 (26.5)				1.02 (0.74,1.42)	
Community-level women's illiteracy							
Low illiteracy	1154 (77.4)	336 (22.6)				1	
High illiteracy	1100 (73.8)	390 (26.2)	0.171	0.017		1.17 (0.85,1.57)	
Community poverty status							
Low	1094 (74.2)	381 (25.8)				1	
High	1160 (77.1)	345 (22.9)	0.125	0.011		0.76 (0.56,1.05)	

^*∗*^Statistically significant variables at 95% confidence interval. Others^+^ = catholic, traditional, and other EDHS categories.

## Data Availability

Data are available online and can be accessed from https://www.measuredhs.com.

## Abbreviations

DIC: Deviance information criterion
AOR: Adjusted odds ratio
DHS: Demographic and Health Survey
EA: Enumeration area
EDHS: Ethiopian Demographic and Health Survey
ICC: Intraclass correlation coefficient
MCSE: Monte Carlo standard error
MCMC: Markov Chain Monte Carlo
PCV: Proportional change in variance
SD: Standard deviation
SNNPR: Southern Nations, Nationalities, and People's Region
SRH: Sexual and reproductive health.

## Supplementary Materials

Supplementary Materials Annex I: convergence-assessment plots for statistically significant parameters.
