# Supplementary material for: Spatial Distribution and Determinants of Nonautonomy on Decision Regarding Contraceptive Utilization among Married Reproductive-Age Women in Ethiopia: Spatial and Bayesian Multilevel Analysis
Source: Nurs Res Pract. 2021 Nov 5;2021:2160922. doi: 10.1155/2021/2160922 (PMC8589474; doi:10.1155/2021/2160922)
Supplement: Supplementary Materials — Annex I: convergence-assessment plots for statistically significant parameters. [file 2160922.f1.pdf]

Annex I: Convergence Assessment plots for Statistically Significant Parameters

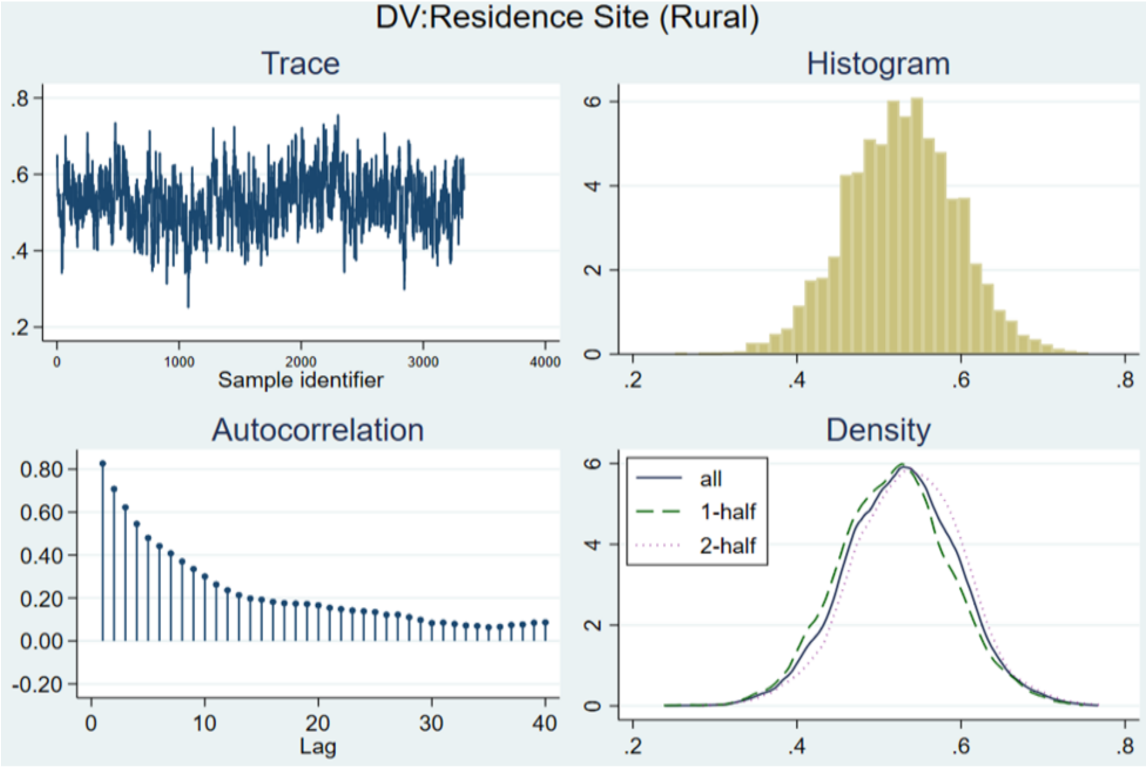

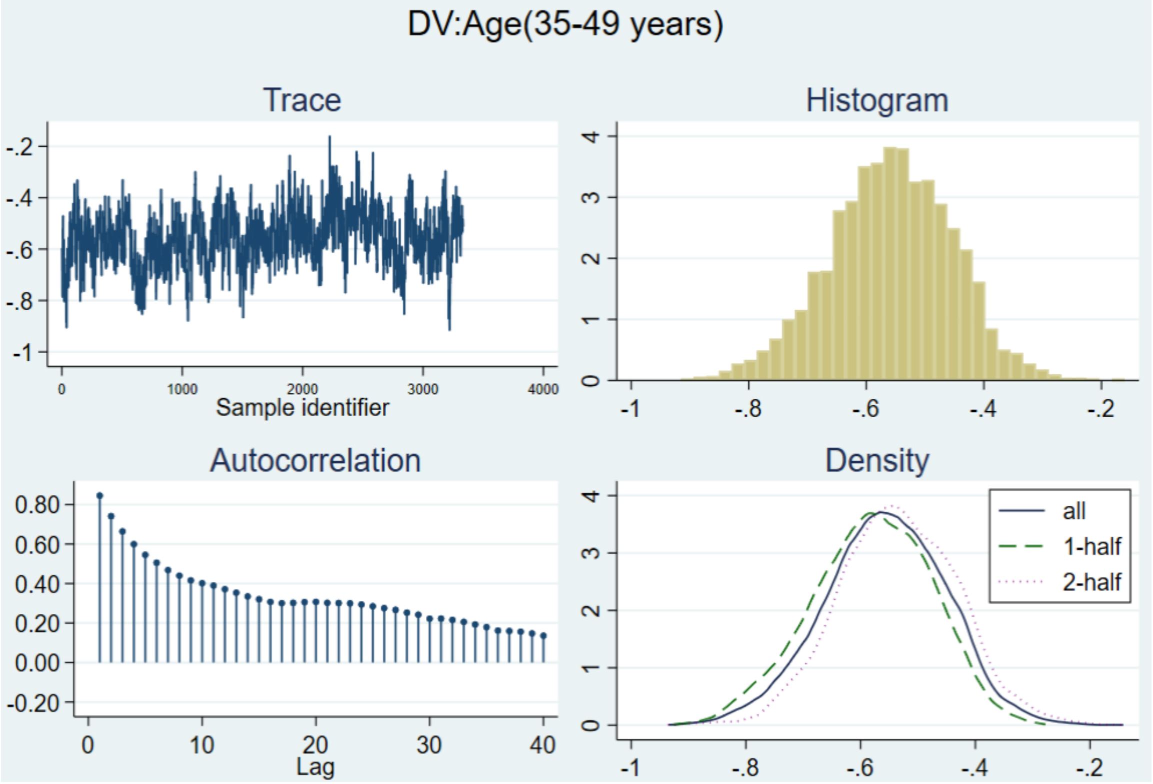

5
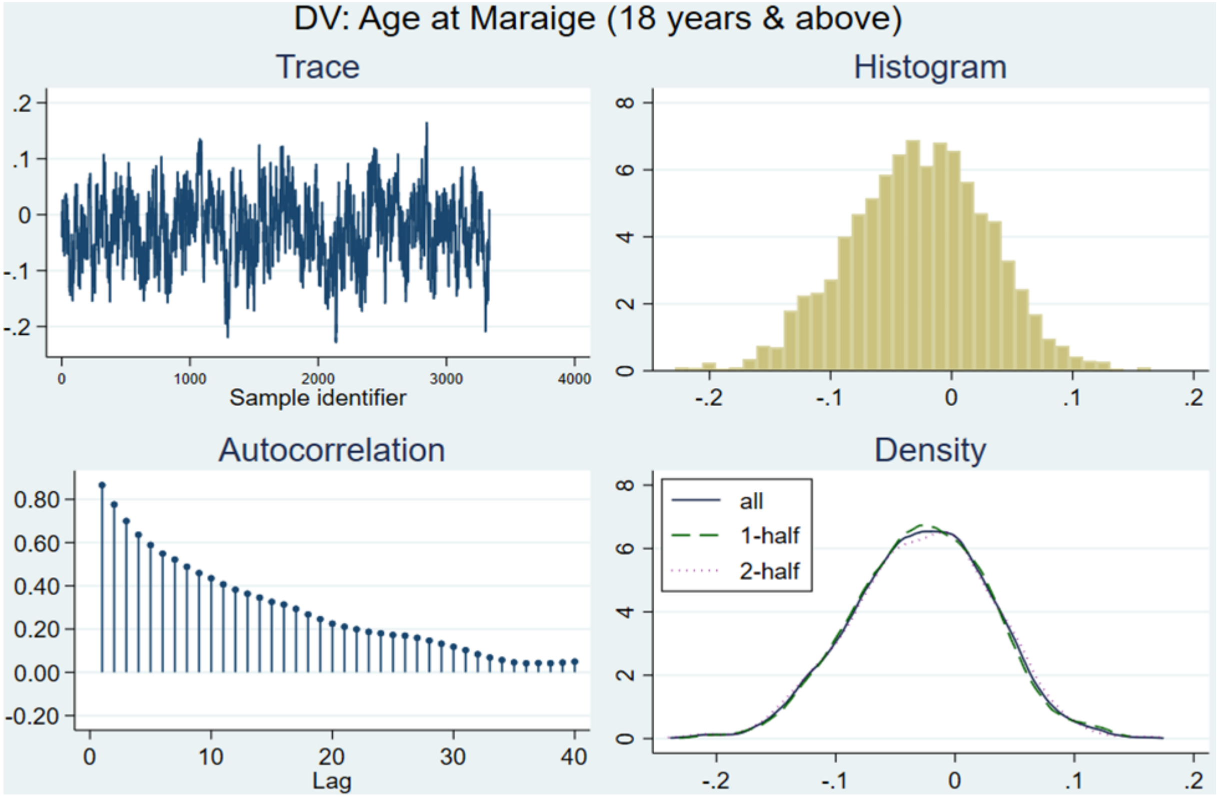
DV: Age at Maraige (18 years & above)
Trace
Sample identifier
Histogram
Autocorrelation
Lag
Density
all
1-half
2-half

6
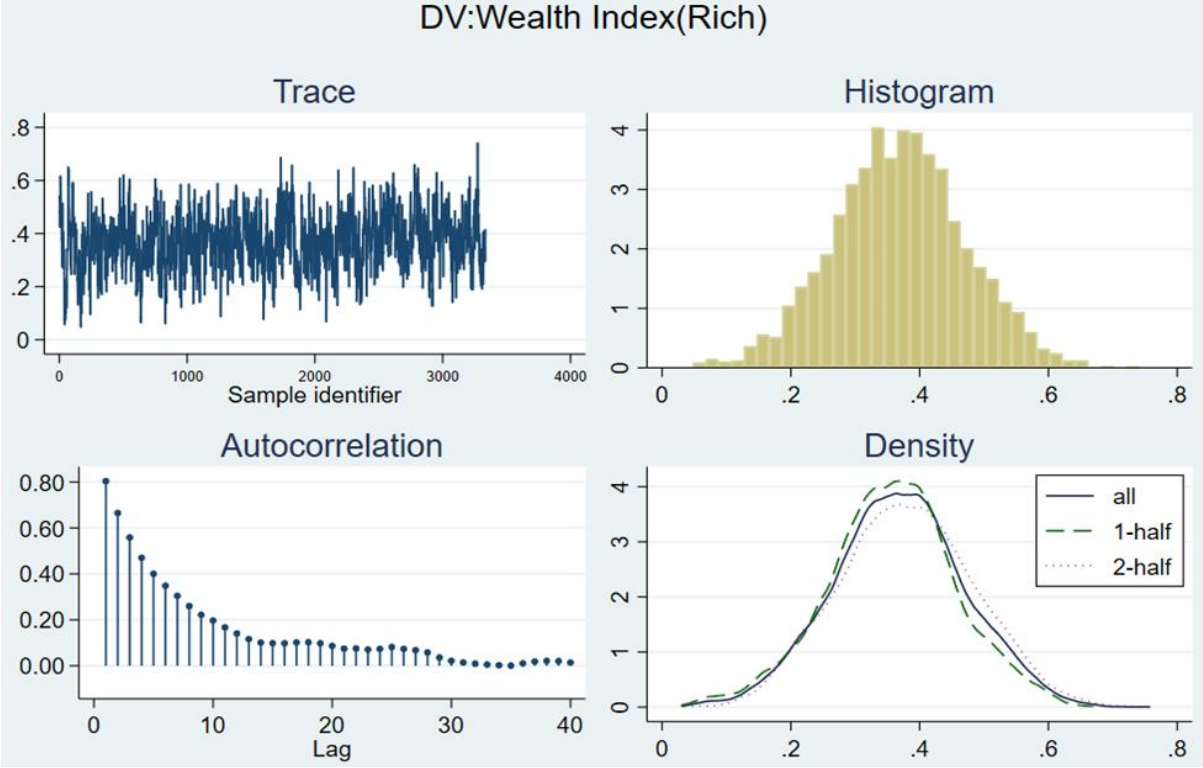
DV:Wealth Index(Rich)
Trace
Sample identifier
Histogram
Autocorrelation
Lag
Density
all
1-half
2-half

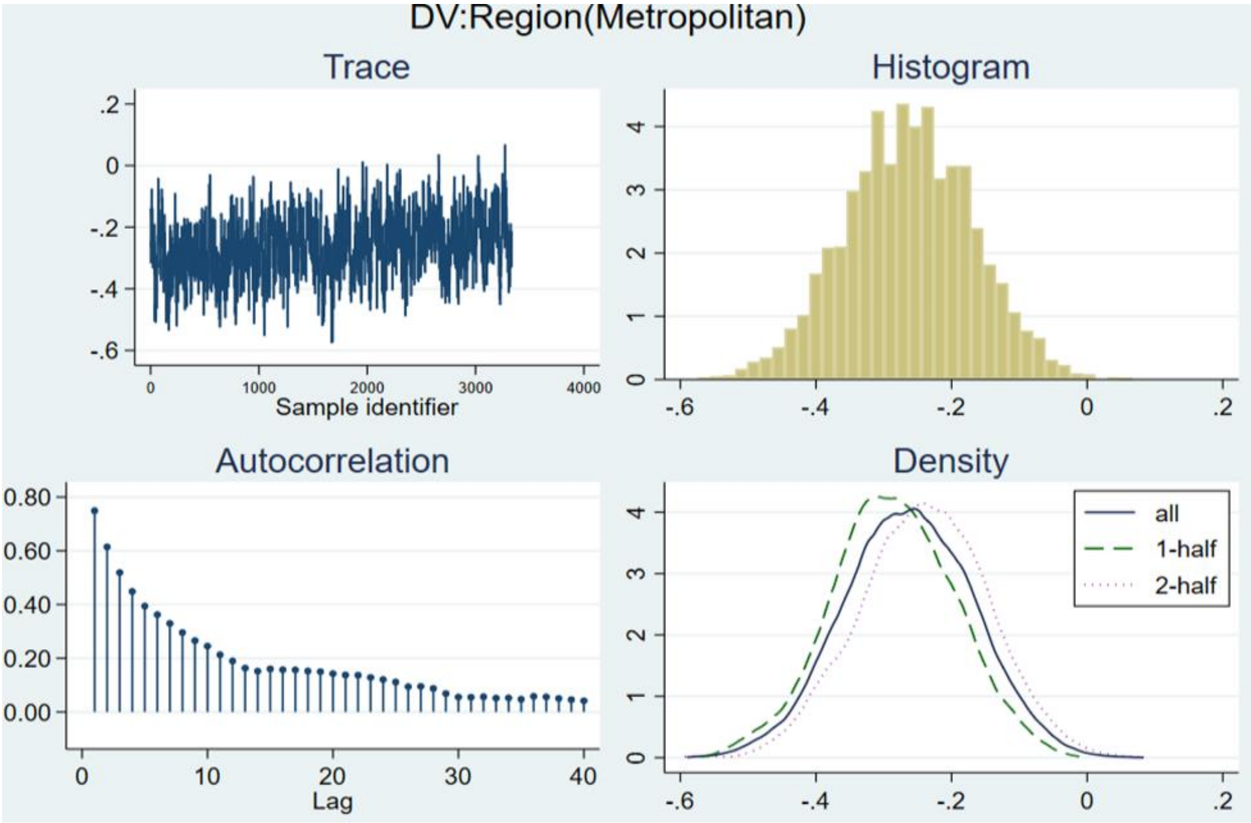

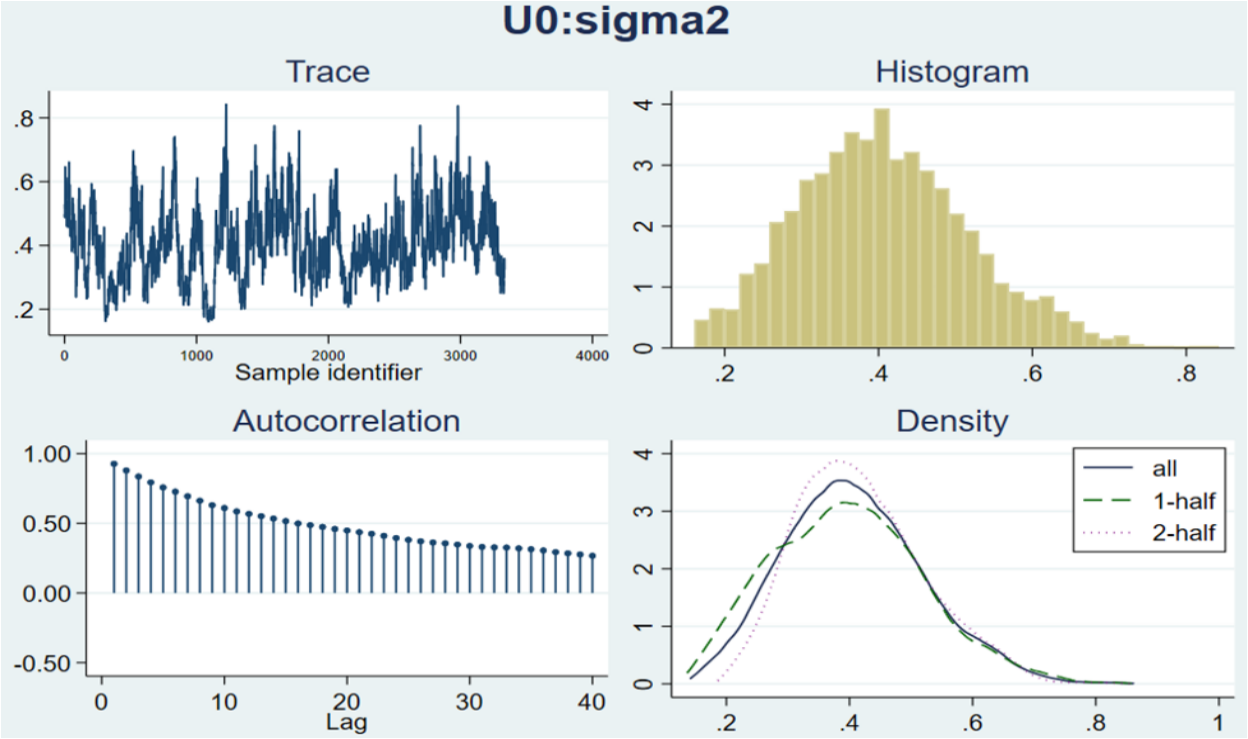

DV: Dependent Variable
